# The Programmable Microbiome: Integrative AI and Multi-Omics Frameworks for Precision T2DM Management

**DOI:** 10.3390/biology15120974

**Published:** 2026-06-22

**Authors:** Barlina Konwar, Kwang-sun Kim

**Affiliations:** Department of Chemistry and Chemistry Institute of Functional Materials, Pusan National University, Busan 46241, Republic of Korea

**Keywords:** Type 2 Diabetes Mellitus, artificial intelligence, pharmaco-microbiomics, precision medicine, digital twin, microbiome engineering

## Abstract

The gut microbiota is recognized as a programmable metabolic organ that governs systemic homeostasis. Recent advances (2023–2025) have pivoted Type 2 Diabetes Mellitus (T2DM) research from a host-centric perspective toward a failure of bidirectional host–microbe metabolic flux. This review evaluates the molecular mechanisms underpinning this shift, focusing on microbial metabolite signaling, virome-mediated modulation, and the emergence of drug–microbiome interactions as critical therapeutic variables. We highlight the transformative role of AI-guided mapping and digital twin simulations in modeling high-resolution metabolic flux and predicting the stability of engineered microbial consortia. By integrating meta-transcriptomics and epigenomics, we characterize the functional plasticity of the microbiome under therapeutic stress. We argue that framing the microbiota as a programmable infrastructure—integrated with AI analytics and metabolic engineering—enables adaptive, real-time interventions. This synthesis offers a blueprint for transitioning from correlative observations toward precision microbiome engineering to achieve sustained metabolic resilience.

## Simple Summary

The trillions of microorganisms living in our gut are no longer seen as passive passengers, but as comprising a vital living factory that helps regulate our entire body’s health. This review addresses the problem that Type 2 Diabetes is traditionally treated only as a human hormone issue, overlooking the vital communication breakdown between our bodies and these microbes. Our objective was to evaluate how new technologies can bridge this gap. We found that by using artificial intelligence and digital twins—virtual computer models of a patient’s gut—scientists can now predict how specially designed “teams” of beneficial microbes will behave during medical treatment. We conclude that the gut can be managed like a programmable system, allowing for precise, real-time adjustments to a patient’s internal environment. This work is valuable to society because it moves us away from one-size-fits-all medicine toward precision engineering of the gut. This shift offers a future where personalized therapies can actively restore health and provide more adaptive, effective long-term care for millions of people living with chronic metabolic diseases.

## 1. Introduction

Type 2 diabetes mellitus (T2DM) is no longer viewed as a multifactorial systemic disorder of insulin resistance and pancreatic β-cell dysfunction but rather as a multifactorial systemic disease shaped by complex interactions among host metabolism, environmental exposures, and the gut microbiota. Although the epidemiological burden of T2DM is well established, its underlying biological mechanisms remain unclear.

Accumulating evidence implicates gut microbiota, a dynamic consortium of bacteria, archaea, viruses, and fungi, as central regulators of metabolic homeostasis. Large-scale systematic reviews have consistently demonstrated associations between gut dysbiosis and T2DM, characterized by reductions in beneficial taxa, such as *Faecalibacterium prausnitzii* and enrichment of pathobionts, including *Escherichia coli–Shigella* [1,2]. Mechanistic studies further revealed that microbial metabolites, notably short-chain fatty acids (SCFAs), bile acid derivatives, and tryptophan catabolites, exert pleiotropic effects on insulin sensitivity, intestinal barrier integrity, incretin signaling, and systemic inflammation [3]. Importantly, recent meta-analytic evidence indicates that microbiome–metabolic associations vary substantially across geographic regions, dietary patterns, and medication exposure, underscoring the need for individualized microbiome profiling in T2DM management [4].

Between 2023 and 2025, the field underwent a notable paradigm shift from descriptive association studies to predictive model-driven frameworks. Advances in multi-omics technologies, including metagenomics, metabolomics, and viromics, coupled with systems pharmacology, have enabled dynamic modeling of host–microbiome metabolic circuits. A recent study has highlighted the importance of strain-level microbial variation, diet–microbe interactions, and virome-mediated horizontal gene transfer in shaping T2DM pathophysiology [5]. The integration of artificial intelligence (AI) and digital twin technologies allows microbiome data, continuous glucose monitoring (CGM), dietary intake, and clinical phenotypes to be fused into personalized computational simulations that support adaptive metabolic care [6].

From a therapeutic perspective, microbiome-informed pharmaco-endocrinology is rapidly expanding. The gut microbiota is increasingly recognized as both a biomarker and a modifiable therapeutic target, shaping individual responses to metformin, glucagon-like peptide-1 receptor agonists (GLP-1 RAs), and dietary interventions. Concurrently, engineered microbial consortia, Clustered Regularly Interspaced Short Palindromic Repeats (CRISPR)–phage hybrid systems, and live biotherapeutic products are entering early-phase human trials as precision microbiome interventions. Collectively, these developments indicate the emergence of microbiome-guided endocrinology, in which metabolic therapies are continuously refined based on patient-specific microbial and metabolic signatures.

Despite these advances, clinical translation remains in the early stages. Key challenges include the standardization of microbial sampling and analytical pipelines; integration of microbiome data into electronic health record systems; development of interpretable AI models; and resolution of regulatory, ethical, and data governance concerns. Given the growing public health burden of diabetes, as recognized by the Centers for Disease Control and Prevention (CDC), and the escalating economic costs associated with its management, innovative and scalable approaches are urgently required [7].

This review critically evaluates the 2023–2025 shift from observational microbiome studies toward an integrative framework that harnesses the gut microbiota as a programmable metabolic axis in T2DM. We highlight the emergence of translational pipelines and AI-driven digital health platforms that transition the gut ecosystem from a passive biomarker to an actively modifiable therapeutic infrastructure. By leveraging system-level approaches—specifically digital twins and synthetic consortia—the field is moving toward real-time, adaptive management of metabolic flux. As we refine the precision of these microbial interventions, the focus must now pivot toward ensuring the long-term ecological stability of engineered communities under therapeutic stress. This evolution marks the transition from broad-spectrum glycemic control to precision-tailored metabolic resilience, where the microbiome serves as a dynamic, responsive interface for personalized medicine.

## 2. Literature Search Strategy and Inclusion Criteria

This review was conducted as a narrative review with a systematic search component, following the methodological principles outlined in the PRISMA 2020 statement where applicable [8]. A structured literature search was performed across four electronic databases: PubMed, Web of Science, Scopus, and Google Scholar. The primary search period spanned January 2023 to March 2026, with selected foundational publications from earlier years included where necessary to establish mechanistic or historical context.

Search queries were constructed using Boolean operators (AND, OR, NOT) combining primary terms—(“gut microbiome” OR “gut microbiota”) AND (“type 2 diabetes” OR “T2DM” OR “insulin resistance” OR “glycemic control”)—with secondary thematic terms applied per section: “artificial intelligence,” “machine learning,” “deep learning,” “digital twin,” “multi-omics,” “metagenomics,” “metabolomics,” “virome,” “mycobiome,” “pharmaco-microbiomics,” “CRISPR,” “bacteriophage,” “live biotherapeutic product,” “engineered microbial consortium,” “flux balance analysis,” “genome-scale metabolic model,” and “continuous glucose monitoring.”

Articles were included if they met all of the following criteria: (i) peer-reviewed original research articles, systematic reviews, or meta-analyses published in English; (ii) focused on human subjects, validated animal models, or computationally validated in silico frameworks; (iii) directly relevant to the mechanistic, computational, or therapeutic dimensions of the T2DM–gut microbiome interface. Articles were excluded if they were: (i) conference abstracts, editorials, or opinion pieces without primary data; (ii) exclusively focused on type 1 diabetes, obesity without T2DM analysis, or non-metabolic conditions; (iii) duplicate reports of the same dataset without new analytical content.

## 3. Global Burden of T2DM

T2DM remains one of the most pressing global health challenges of the 21st century and is characterized by a rapidly increasing prevalence, substantial economic burden, and persistent health inequities. According to the CDC report, an estimated 38.4 million individuals in the United States alone, approximately 11.6% of the population, currently live with diabetes, of whom 90–95% have T2DM [7,9]. Alarmingly, nearly 115.2 million U.S. adults meet the criteria for prediabetes, with the majority unaware of their condition, placing them at heightened risk for progression to overt disease [10].

**Figure 1:**
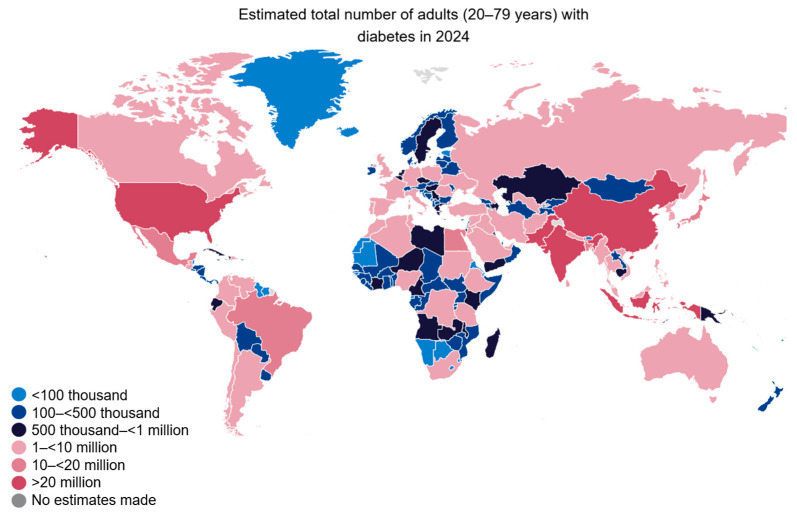
Estimated total number of adults (20–79 years) with diabetes in 2024. Reprinted from [10], with permission.

Thus, the global burden is even more significant. According to the International Diabetes Federation (IDF), approximately 589 million adults aged 20–79 years, nearly one in nine worldwide, are currently living with diabetes, which is projected to increase to 853 million by 2050 [10,11] (Figure 1). Notably, nearly three-quarters of diabetes cases occur in low- and middle-income countries, where access to preventive care, diagnostics, and long-term disease management remains limited, exacerbating disparities in outcomes [11,12]. Diabetes also imposes a substantial mortality burden, accounting for an estimated 3.4 million deaths globally by 2024, with approximately one death in every nine seconds [11].

The economic consequences are profoundly equal. The global direct healthcare expenditure attributable to diabetes reached USD 966 billion by 2021, representing a 316% increase over the past 15 years [11], and rose further to an estimated USD 1.015 trillion by 2024 [11]. Diabetes remains a leading cause of cardiovascular disease, chronic kidney disease, blindness, and premature mortality, significantly diminishing both lifespan and quality of life.

Demographic aging, rapid urbanization, and lifestyle transitions characterized by sedentary behavior and energy-dense diets strongly suggest that T2DM prevalence will continue to rise in the coming decades. Although prevention strategies centered on healthy nutrition, physical activity, and early detection have the potential to avert a substantial proportion of new cases, their equitable and sustained implementation remains a formidable challenge across diverse populations.

Therefore, innovative approaches are urgently needed. The integration of AI-enabled risk modeling, digital health monitoring, and microbiome-informed interventions offers a promising pathway for the precise prevention and personalized management of T2DM. By enabling earlier risk stratification, continuous metabolic monitoring, and individualized therapeutic adaptation, these emerging strategies have the potential to shift diabetes care from reactive disease management to proactive data-driven prevention and long-term metabolic resilience.

## 4. The Gut Microbiota as a Programmable Metabolic Axis

The gut microbiota has emerged as a dynamic, endocrine-like organ that exerts pervasive control over host metabolism, glucose homeostasis, and inflammatory tone. Rather than constituting a passive assemblage of commensal microorganisms, the intestinal ecosystem operates as an integrated metabolic signaling network that continuously interfaces with the hepatic, adipose, pancreatic, and neuroendocrine systems. Accumulating mechanistic and clinical evidence implicates microbial composition, functional capacity, and metabolite output as key determinants of insulin sensitivity, β-cell integrity, and chronic low-grade inflammation, the central pathophysiological triad underpinning T2DM [1].

### 4.1. Ecological Architecture and Functional Dysbiosis

In healthy individuals, the gut microbiome is dominated by Firmicutes, Bacteroidetes, Actinobacteria, and Proteobacteria, which maintain a balanced fermentative environment that supports epithelial integrity and metabolic equilibrium. In contrast, multi-omics studies have demonstrated that individuals with T2DM exhibit significant microbial dysbiosis. This dysbiosis is characterized by a marked reduction in butyrate-producing taxa, particularly *Faecalibacterium prausnitzii* and *Roseburia intestinalis*, which play important roles in maintaining the gut barrier and anti-inflammatory regulation [13,14,15,16,17]. Simultaneously, T2DM is associated with the expansion of opportunistic and pro-inflammatory species, including *Ruminococcus gnavus* and *Escherichia coli–Shigella*, which have been linked to mucus degradation, endotoxin production, and inflammatory signaling [14]. Metagenomic and functional profiling further revealed a shift in microbial metabolic capacity, marked by impaired carbohydrate fermentation, reduced expression of genes involved in SCFA biosynthesis, and enrichment of lipopolysaccharide (LPS) biosynthetic pathways. These functional alterations are strongly associated with metabolic endotoxemia, systemic inflammation, and insulin resistance. However, the reproducibility and universality of these microbial signatures across geographically and clinically diverse populations remain subjects of ongoing investigation.

A schematic summary of the principal taxonomic and functional microbial alterations associated with T2DM is presented in Table 1, which highlights the conserved dysbiotic signatures across diverse cohorts.

#### Cross-Cohort Reproducibility and Geographic Heterogeneity

Despite the associations described above, careful evaluation of their reproducibility across populations and study designs remains essential. A recent meta-analysis of 16S rRNA amplicon-based studies demonstrated substantial inter-study heterogeneity in T2DM-associated microbial signatures, with concordance strongly influenced by geographic origin, dietary patterns, sequencing platforms, and the selected 16S hypervariable regions [4]. Foundational shotgun metagenomic studies conducted in European cohorts identified members of the *Clostridiales* and *Lactobacillaceae* as major discriminatory taxa [18], whereas a contemporaneous large-scale Chinese metagenome-wide association study instead highlighted *Clostridium hathewayi* and *Eggerthella lenta* [19]. South Asian and African cohorts have demonstrated additional divergence in taxa-level associations, supporting the major contribution of diet and environmental exposures relative to host genetics in shaping gut microbial composition [20].

Critically, a meta-analysis of 28 gut microbiome case–control studies across multiple diseases demonstrated that many reported disease associations represent population-specific signals rather than universal biomarkers, with taxonomic effect sizes heavily confounded by lifestyle, antibiotic history, and medication exposure [21]. In the T2DM context specifically, multiple microbiome alterations initially attributed to disease have been subsequently shown to reflect confounding by metformin treatment, obesity, or age, rather than constituting independent pathophysiological signals [22].

A further methodological concern is that relative abundance profiling using 16S amplicon sequencing—the dominant approach across published T2DM cohorts—is susceptible to compositional biases and cannot distinguish changes in absolute microbial load from shifts in community proportions, raising concerns about causal inference [23]. Collectively, these considerations indicate that while the core dysbiotic signatures described in Table 1 represent the most consistently replicated associations in the literature, their magnitude, taxonomic specificity, and causal relevance vary substantially across populations and study designs. Multi-cohort validation using harmonized shotgun metagenomics and absolute quantification approaches remains an unmet methodological priority.

### 4.2. Metabolite-Mediated Host Signaling

Microbial metabolites function as potent bioactive messengers that couple gut ecosystem dynamics with host metabolism and immune physiology. SCFAs, including acetate, propionate, and butyrate, are generated through the microbial fermentation of dietary fibers and exert pleiotropic effects on host metabolism. These metabolites regulate hepatic gluconeogenesis, adipocyte differentiation, and incretin hormone secretion through activation of G-protein-coupled receptors (GPR41 and GPR43) and epigenetic modulation via histone deacetylase inhibition. Depletion of SCFAs compromises intestinal barrier integrity, promotes endotoxin translocation, and disrupts insulin signaling pathways, thereby contributing to metabolic inflammation and insulin resistance.

In parallel, microbial biotransformation of primary bile acids yields secondary bile acids, which act as signaling molecules that influence glucose and lipid homeostasis. These metabolites engage nuclear and membrane-bound receptors, including farnesoid X receptor (FXR) and Takeda G protein-coupled receptor 5 (TGR5), to regulate energy expenditure, insulin sensitivity, and enterohepatic signaling [5]. Beyond lipid-derived metabolites, microbial tryptophan catabolism generates indole derivatives, such as indole-3-propionic acid and indole-3-acetic acid, which exert antioxidant and anti-inflammatory effects while preserving pancreatic β-cell viability.

Consistent with these mechanistic insights, multi-omics analyses have demonstrated that reduced circulating levels of indole metabolites are independently associated with elevated homeostasis model assessment of insulin resistance indices and heightened inflammatory markers in individuals with newly diagnosed T2DM [3]. Collectively, these findings underscore the central role of microbiota-derived metabolites as molecular intermediaries that link the gut microbial composition to host metabolic dysfunction.

### 4.3. Inflammation, Barrier Integrity, and Immune Crosstalk

Gut microbial dysbiosis compromises intestinal epithelial barrier integrity, facilitating the translocation of microbial-associated molecular patterns, including LPS and peptidoglycan, into systemic circulation. This state of metabolic endotoxemia activates innate immune pathways via Toll-like receptors (TLR2 and TLR4), triggering nuclear factor kappa B (NF-κB) signaling and downstream pro-inflammatory cytokine cascades, notably tumor necrosis factor-α and interleukin-6, which collectively impair insulin signaling and promote insulin resistance. Experimental restoration of *Akkermansia muciniphila* or supplementation with SCFAs in preclinical models has been shown to reinforce epithelial tight junction integrity, attenuate inflammatory signaling, and improve glucose tolerance and insulin sensitivity [17,24]. Recent single-cell immune transcriptomic analyses have revealed that gut dysbiosis reshapes mucosal immune landscapes, particularly by skewing the balance between pro-inflammatory T helper 17 and regulatory T cells, thereby sustaining the chronic low-grade inflammation characteristic of T2DM.

### 4.4. Microbiota–Drug and Host Feedback Loops

The gut microbiota functions not only as a responder to host metabolic status but also as an active determinant of pharmacological efficacy and tolerability. Metformin, the first-line oral therapy for T2DM, exerts a substantial glucose-lowering effect through microbiome-mediated mechanisms, including enhanced SCFA production and modulation of the bile acid signaling pathways. Recent metagenomic analyses demonstrated that metformin treatment increased the abundance of *A. muciniphila* and enriched microbial glyoxylate cycle genes, contributing to improved regulation of hepatic gluconeogenesis [24]. Conversely, microbial enzymes, most notably β-glucuronidases, can alter drug bioavailability by reactivating glucuronidated compounds or generating toxic metabolites, underscoring the bidirectional feedback between gut microbial composition and host pharmacokinetics. These reciprocal interactions highlight the microbiota as a critical modifier of the therapeutic response in T2DM.

## 5. Viriome, Mycobiome, and Host–Microbe Crosstalk in T2DM

Although most metabolic disease research has historically centered on bacterial communities, the gut virome and mycobiome are highly dynamic and functionally influential components of the intestinal ecosystem. Bacteriophages, eukaryotic viruses, and commensal fungi actively shape the microbial community structure through predation, horizontal gene transfer, and metabolic reprogramming, while simultaneously engaging in host immune pathways. Emerging multi-kingdom metagenomic and systems-level analyses indicate that perturbations in viral and fungal ecology not only co-occur with bacterial dysbiosis in T2DM but may also precede and amplify it, thereby contributing to metabolic inflammation and disease progression [13].

### 5.1. The Gut Virome as a Metabolic Modulator

The gut virome, composed predominantly of bacteriophages, is estimated to outnumber bacterial cells by up to an order of magnitude, and represents a major regulatory layer of the intestinal ecosystem. Through selective predation, lysogenic conversion, and horizontal gene transfer, bacteriophages exert top-down control over bacterial community structure, dynamically reshaping microbial metabolic capacity and influencing SCFA biosynthesis, bile acid transformation, and host immune signaling [13].

Longitudinal meta-viromic analysis revealed pronounced virome alterations in T2DM patients. Patients with newly diagnosed diseases exhibit a reduced richness of Caudoviricetes and a relative expansion of temperate phages targeting Enterobacteriaceae, changes that correlate with increased fecal LPS activity, and elevated fasting glucose concentrations [1]. Mechanistically, lysogenic phages contribute auxiliary metabolic genes that modulate bacterial carbohydrate and lipid metabolism, illustrating how viral genetic flux can recalibrate microbial–host energy exchange and metabolic inflammation.

**Figure 2:**
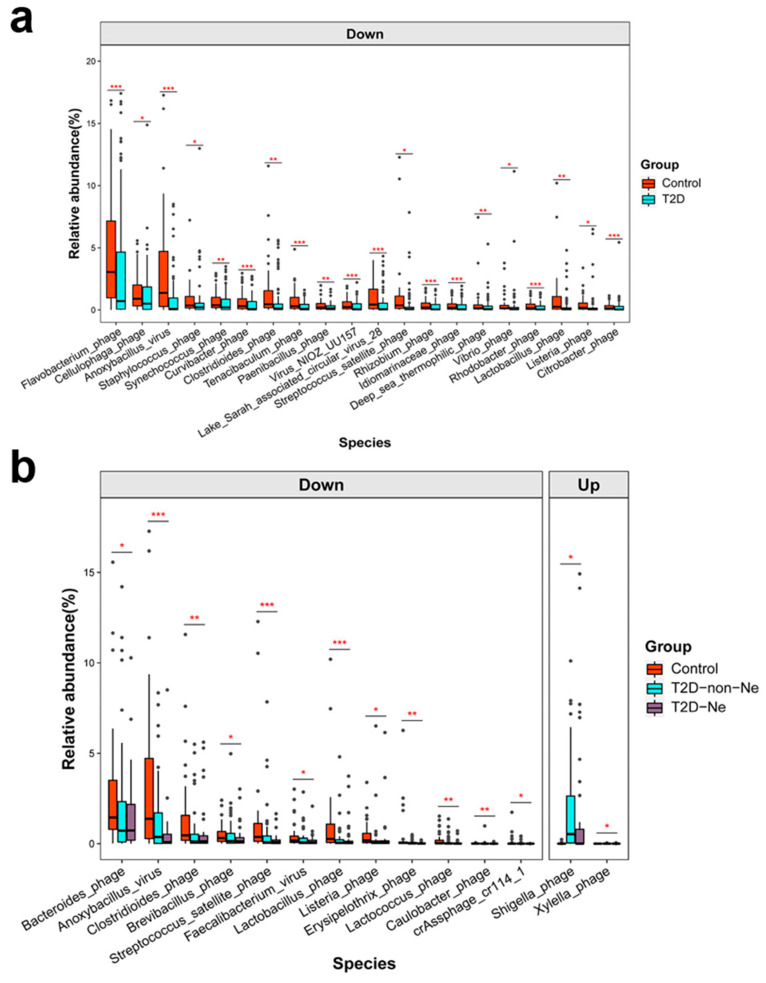
Gut virome remodeling and its functional links to metabolic dysfunction in T2DM. (**a**) Differential viral species between T2DM and healthy controls, identified using MaAsLin2 analysis and adjustment for confounders, including age, gender and diabetic nephropathy (DN). Only the top 20 most abundant species are shown. (**b**) Differential viral species between T2DM-Ne and healthy controls, identified using MaAsLin2 analysis with adjustment for age and gender. In both (**a**,**b**), statistical significance was defined as *p* < 0.05 (* *p* < 0.05, ** *p* < 0.01, *** *p* < 0.001). Individual data points (dots) represent the relative abundance (%) of each species in study participants. In the box plots, boxes represent the interquartile range (25th to 75th), with the center line indicating the median. Reprinted from [26] under the Creative Commons Attribution-NonCommercial 4.0 International License (CC BY-NC 4.0).

Multivariable association analysis using multivariable association with linear model 2 [25] further demonstrated robust links between T2DM-associated virome features and metabolic parameters, after adjusting for relevant clinical covariates. In total, 81 viral species were significantly altered in individuals with T2DM, including a marked reduction in several bacteriophages against *Cellulophaga* and *Flavobacterium* [26]. As illustrated in Figure 2a, these alterations reflect global virome dysbiosis, which is characterized by shifts in viral diversity and phage composition. The functional relevance of these changes is further underscored in Figure 2b, which shows that virome–metabolic associations are more pronounced in T2DM patients with diabetic neuropathy than in those without neuropathy, linking specific viral taxa and phage-encoded metabolic genes to disease severity and neuropathy-associated metabolic dysfunctions.

In addition to bacteriophages, eukaryotic enteric viruses, including norovirus and enteroviruses, may indirectly influence glucose metabolism by promoting mucosal inflammation and disrupting epithelial homeostasis. Experimental infections in gnotobiotic models have been shown to impair tight junction integrity and amplify systemic cytokine responses, thereby predisposing the host to insulin resistance and metabolic dysregulation [5].

### 5.2. Phage–Bacterium–Host Signaling Networks

Recent studies have revealed intricate and dynamic signaling interactions among bacteriophages, bacterial communities, and host immune sensors, forming a tripartite communication network that extends beyond microbial community regulation to influence the host systemic physiology. Phage-derived nucleic acids can be sensed by host innate immune receptors, including TLR3 and TLR9, as well as the cyclic GMP-AMP synthase stimulator of interferon gene-associated pathway, leading to type I interferon responses that modulate systemic inflammatory tone and metabolic homeostasis.

Conversely, bacterial populations actively regulate their susceptibility to phage infection through quorum sensing mechanisms. Quorum-sensing molecules can alter phage adsorption, replication, and lysogenic–lytic switching, thereby maintaining the ecological stability within the gut microbiome. This bidirectional signaling suggests that perturbations of the virome may function as upstream drivers of bacterial dysbiosis, metabolic endotoxemia, and chronic inflammatory stress in T2DM [24].

From a therapeutic perspective, phage engineering has emerged as a precise strategy for selectively targeting the bacterial drivers of dysmetabolism. Preclinical studies have demonstrated that CRISPR-equipped bacteriophages directed against pro-inflammatory taxa, such as *Enterobacter cloacae* and *Desulfovibrio piger*, can reduce intestinal LPS burden, attenuate systemic inflammation, and improve insulin sensitivity in murine models of diet-induced diabetes [1]. Collectively, these findings suggest phage–bacterium–host signaling networks are both mechanistic contributors to T2DM pathogenesis and are promising targets for next-generation microbiome therapeutics.

### 5.3. The Mycobiome: Fungal–Bacterial–Immune Crosstalk

Although they constitute less than 0.1% of the total gut microbial biomass, the gut mycobiome exerts a disproportionate influence on host immunity and metabolic regulation. Fungal communities are typically dominated by *Candida*, *Malassezia*, and *Saccharomyces* species, which participate in bile acid transformation, carbohydrate fermentation, and the modulation of SCFA availability. In T2DM, mycobiome dysbiosis is characterized by an expansion of *Candida albicans* and depletion of beneficial fungi such as *Saccharomyces boulardii*, alterations that correlate with impaired glucose tolerance, increased intestinal permeability, and mucosal inflammation [1].

Fungal cell wall components, including β-glucans and mannans, are recognized by host pattern-recognition receptors, such as dectin-1 and mannose receptor, triggering NF-κB-dependent inflammatory signaling and cytokine production [27]. Elevated circulating antifungal IgG titers in individuals with T2DM further support chronic low-grade fungal translocations and immune activation. Additionally, in vitro co-culture studies provided mechanical insights, demonstrating that *Candida*-associated changes in the microbial environment can suppress the growth of *A. muciniphila* and *Bifidobacterium* species, thereby exacerbating bacterial dysbiosis and metabolic imbalance [28].

### 5.4. Multi-Kingdom Interactions and Systemic Effects

The concept of a multi-kingdom microbiome emphasizes interconnected and reciprocal interactions between bacteria, bacteriophages, fungi, and the host. Advances in integrative meta–multi-omics approaches—combining viromics, mycobiomics, metagenomics, and metabolomics—have enabled the reconstruction of cross-domain ecological and functional networks within the gut. Network-based analyses have revealed that the phage-mediated depletion of butyrate-producing bacteria can indirectly promote fungal overgrowth, establishing a self-reinforcing inflammatory loop that links viral, bacterial, and fungal dysbiosis.

At the systemic level, these cross-kingdom perturbations influence hepatic gluconeogenesis, adipose tissue inflammation, and gut–brain neural signaling, collectively shaping metabolic phenotypes and disease heterogeneity in T2DM. A recent commentary proposed that the virome–mycobiome axis functions as an immune amplifier within the gut–liver–brain axis, magnifying inter-individual variability in inflammatory tone, insulin sensitivity, and disease progression [24]. Table 2 summarizes these multi-kingdom interactions and their systemic effects on host metabolic pathways.

## 6. Pharmaco-Microbiomics and Drug–Microbiome Interactions

The concept of pharmaco-microbiomics, the study of how gut microorganisms modulate drug pharmacokinetics, efficacy, and toxicity, has emerged as a central framework for explaining inter-individual variability in therapeutic responses to T2DM treatments. The gut microbiota not only directly metabolizes xenobiotics, but also reshapes the host physiological pathways that govern drug absorption, bioavailability, and systemic exposure. Conversely, many anti-diabetic agents exert microbiome-modifying effects by restructuring microbial composition and metabolic output. This establishes a dynamic bidirectional feedback loop in which microbial activity can either potentiate or attenuate pharmacological efficacy, ultimately influencing clinical outcomes [13].

### 6.1. Microbial Modulation of Drug Metabolism

The gut microbiome encodes an extensive and chemically diverse enzymatic repertoire capable of transforming xenobiotics into bioactive, inactive, or toxic metabolites, thereby exerting a critical influence on drug pharmacokinetics and pharmacodynamics. Microbial enzymes, including reductases, hydrolases, and de-hydroxylases, mediate bio-transformations within the intestinal lumen, thereby directly shaping drug bioavailability, systemic exposure, and therapeutic efficacy. Canonical examples include *Eggerthella lenta*, which inactivates digoxin through cardiac glycoside reductase activity, and *Clostridium scindens*, which converts corticosteroids into androgenic metabolites, highlighting the capacity of the microbiome to remodel host drug responses at a systemic level [5].

In the context of diabetes, emerging evidence suggests that analogous microbiome-mediated reactions modulate the metabolism of antidiabetic agents, including biguanides, sulfonylureas, and incretin-based therapies, thereby influencing inter-individual variability in drug exposure, efficacy, and adverse effects. These findings suggest that the gut microbiome is a previously underappreciated determinant of therapeutic response and underscores its potential as a target for precision pharmacotherapy in metabolic diseases.

### 6.2. Metformin: A Microbiome-Dependent Drug

Among anti-diabetic therapies, metformin provides the most compelling example of bidirectional host–microbiome–drug interactions. Metformin, once regarded primarily as a hepatic activator of AMP-activated protein kinase, is now recognized as a potent modulator of the gut microbial ecology and function. Accumulating evidence demonstrates that metformin treatment consistently enriches beneficial taxa, including *A. muciniphila* and *Bifidobacterium adolescentis*, enhances SCFA production, and reshapes bile acid pools through the FXR and TGR5 signaling pathways. Together, these microbiome-driven effects converge to improve insulin sensitivity, suppress hepatic gluconeogenesis, and substantially contribute to the glucose-lowering efficacy of metformin [30].

Longitudinal multi-omics analyses integrating metagenomic sequencing with plasma metabolomics further reinforce the microbiome dependence of the metformin response. Individuals classified as metformin responders exhibit selective enrichment of butyrate-producing bacterial species, along with reduced circulating levels of imidazole propionate, a microbial metabolite causally linked to impaired insulin signaling. These findings underscore the potential of gut microbiome signatures as predictive biomarkers of metformin efficacy and highlight the microbiome as a modifiable determinant of therapeutic response in T2DM [1].

### 6.3. GLP-1 Receptor Agonists, SGLT2 Inhibitors, and Beyond

Emerging data suggest that GLP-1 RAs and sodium–glucose cotransporter 2 (SGLT-2) inhibitors also exert microbiome-mediated effects. GLP-1 RAs increase intestinal transit time and alter bile acid circulation, indirectly reshaping the microbial composition. In murine and human studies, liraglutide therapy enriched *Akkermansia* and *Lactobacillus* while suppressing *Desulfovibrio*, paralleling improvements in glycemic and inflammatory markers [3].

SGLT2 inhibitors, including dapagliflozin, further modify the intestinal ecosystem by increasing luminal glucose availability and altering gut pH, thereby creating a metabolic niche that favors SCFA-producing microorganisms [31]. The resulting increase in SCFA biosynthesis is associated with enhanced insulin sensitivity, improved gut barrier integrity, and attenuation of low-grade inflammation. Integrative microbiome-intervention reviews suggest that drug-induced microbial shifts may be potentiated by adjunctive prebiotic, probiotic, postbiotic, or synbiotic strategies, but these combinations require prospective testing before clinical adoption [32,33].

### 6.4. Bidirectional Feedback and Systems Pharmacology

The interaction between pharmacological agents and the gut microbiota is inherently bidirectional. While therapeutic drugs remodel the microbial community structure and metabolic output, the microbiota reciprocally modulates systemic drug disposition through the production of signaling metabolites, including SCFAs, secondary bile acids, and indole derivatives, which regulate host pathways, such as hepatic cytochrome P450 enzyme expression and activity. Systems pharmacology approaches that integrate microbial genomic profiles with host transcriptomic and metabolomic data have begun to delineate these complex feedback circuits, enabling the identification of network nodes predictive of therapeutic responses, interindividual variability, and adverse drug reactions [34].

A particularly well-characterized mechanism involves microbial reactivation of glucuronidated drugs and drug metabolites within the intestinal lumen. Bacterial β-glucuronidases can deconjugate glucuronide moieties added during hepatic phase II metabolism, restore pharmacological activity, or generate toxic compounds locally. This process has been implicated in drug-induced gastrointestinal toxicity, including metformin-associated intolerance, where excessive microbial β-glucuronidase activity exacerbates luminal drug exposure. Targeted inhibition of these microbial enzymes without broadly disrupting the gut microbiota has emerged as a promising strategy for improving drug tolerability while preserving glycemic efficacy, underscoring the translational potential of microbiome-informed pharmacological interventions.

### 6.5. Precision Pharmaco-Microbiomics

The convergence of artificial intelligence and multi-omics technologies enables the individualized prediction of drug efficacy based on gut microbial composition and functional metabolic capacity. Deep-learning frameworks trained on integrated metagenomic, metabolomic, and pharmacokinetic datasets have demonstrated superior performance in forecasting glycemic responses to antidiabetic therapies, including metformin and GLP-1 RAs, compared with conventional clinical predictors alone [1]. These advances provide a foundation for microbiome-informed dosing algorithms and rational combination strategies to optimize the therapeutic efficacy while minimizing adverse effects. Beyond predictive modeling, pharmaco-microbiomics enables dynamic treatment adaptation through ecological modulation of the gut microbiota, positioning microbial features as actionable determinants of drug response.

For pharmaco-microbiomic AI frameworks to be clinically useful, pharmacological covariates must be modeled explicitly rather than treated as background noise. Required variables include drug dose, formulation, treatment du-ration, adherence, renal and hepatic function, concomitant medications, gastrointestinal transit time, microbial enzyme activities, host pharmacokinetic/pharmacodynamic exposure, and adverse-event profiles. Where relevant, models should also incorporate peripheral accumulation of active metabolites and dose-dependent toxicity, because microbiota-derived or microbiota-reactivated metabolites may affect tissues beyond the intestinal lumen. These variables are necessary for distinguishing microbial predictors of therapeutic benefit from microbial signatures that merely reflect drug exposure or intolerance [34].

**Figure 3:**
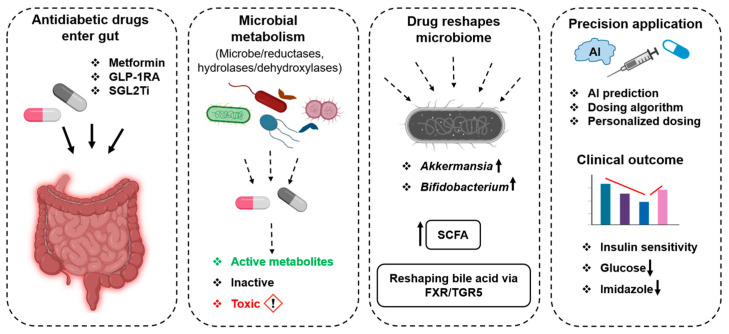
Graphical representation of pharmaco-microbiomics and drug–microbiome interactions, highlighting their pathway toward precision clinical use. Solid arrows indicate direct drug entry/transit whereas dashed arrows represent microbial metabolic transformation or downstream regulatory effects on the microbiome. Up/down arrows (↑/↓) denote increased or decreased abundance/levels. Created with BioRender.com.

Collectively, this emerging paradigm represents a cornerstone of algorithmic precision endocrinology, in which computational analytics, systems pharmacology, and microbiome engineering converge to tailor therapies to each patient’s unique microbial and metabolic fingerprints. Figure 3 presents a conceptual overview of pharmaco-microbiomics and drug–microbiome interactions, illustrating the mechanisms by which microbial factors influence drug response and how these insights translate into precision clinical applications.

## 7. Toward Adaptive and Programmable Microbiome Medicine into T2DM

### 7.1. The Programmable Microbiome Model

Advances in AI and integrative multi-omics have catalyzed the transition from descriptive microbiome analyses to predictive and programmable metabolic modeling. Traditional analysis primarily catalogs taxonomic differences; however, such static snapshots fail to capture the dynamic and context-dependent behavior of the gut ecosystem in metabolic disorders like T2DM. In contrast, emerging computational frameworks explicitly model the gut microbiota as a dynamic and adaptive system whose functional state can be predicted and optimized for therapeutic interventions [35,36].

Deep learning models trained on metagenomic, metabolomic, and clinical data now enable accurate prediction of postprandial glycemic responses and microbial signatures associated with therapeutic efficacy [1,35,36,37]. These frameworks integrate host genetic, dietary, microbial, and pharmacological layers into a system-level framework for personalized management. This capability underpins emerging digital twin platforms for precision endocrinology [29], reframing the gut microbiota from a passive biomarker to a programmable metabolic actuator.

### 7.2. Digital Twin Systems for T2DM

A digital twin in biomedicine is a continuously updated, patient-specific computational model designed to replicate an individual’s physiological and metabolic state using longitudinal biological and clinical data [29]. In the context of T2DM, microbiome-integrated digital twins combine microbial, metabolic, dietary, and clinical information into dynamic predictive systems capable of simulating glycemic responses to dietary interventions, pharmacotherapy, or microbiome-targeted modulation prior to clinical implementation [38].

Digital twin platforms integrate genome-scale metabolic models (GEMs), community flux balance analysis frameworks such as microbial community modeling (MICOM) and machine-learning approaches including long short-term memory (LSTM) and gradient-boosting algorithms to analyze microbiome dynamics, metabolite exchange, and individualized glycemic responses [39,40,41,42]. These systems are increasingly linked with continuous glucose monitoring, wearable sensors, dietary records, and multi-omics datasets to enable real-time physiological monitoring.

Core state variables typically include microbial abundance and functional pathways, SCFAs, bile acids, tryptophan metabolites, lipopolysaccharides, CGM-derived glycemic metrics, HbA1c, insulin resistance indices, inflammatory biomarkers, dietary composition, medication exposure, and physical activity patterns [42].

Validation strategies currently include cross-validation analyses, external cohort testing, ex vivo fermentation calibration, and retrospective comparison of predicted versus observed glycemic outcomes [6]. However, prospective multicenter randomized validation of microbiome-integrated digital twins in T2DM has not yet been achieved and remains a major requirement for clinical translation [38].

To operationalize these AI-enabled pharmaco-microbiomic frameworks, model development should use harmonized metagenomic, metabolomic, dietary, CGM, pharmacological, and clinical variables, followed by batch-effect correction, predefined training/validation/test splits, and benchmarking against conventional clinical predictors [41,43]. Relevant methods include gradient boosting, random forests, regularized regression, temporal models for CGM time-series data, and graph- or constraint-based models for microbial metabolic exchange [36,43]. Model performance should be reported using task-appropriate metrics, including the area under the receiver operating characteristic curve (AUROC) for classification, root-mean-square error (RMSE) for glycemic prediction, calibration curves, decision-curve analysis, and interpretable feature-attribution methods such as SHapley Additive exPlanations (SHAP) [44]. Because microbiome datasets are high-dimensional, cohort-specific, and vulnerable to processing bias, overfitting, medication confounding, and population-specific microbial signatures, nested cross-validation, external geographic validation, medication-stratified analysis, and prospective testing are required before clinical use [25,45,46].

However, current microbiome-integrated digital twin studies in T2DM remain highly heterogeneous and are limited by several methodological and translational challenges. Many existing platforms rely on proprietary computational algorithms, short follow-up durations, and single-center or commercial real-world cohorts, thereby restricting reproducibility and broader clinical generalizability. In addition, predictive accuracy may be affected by inter-individual population variability, microbiome batch effects, incomplete wearable sensor data, dietary heterogeneity, and unmeasured factors such as medication adherence and lifestyle behaviors. The high dimensionality and dynamic nature of microbiome datasets further increase the risk of model overfitting and reduced external validation performance across independent cohorts. Consequently, although microbiome-integrated digital twins show considerable promise for personalized metabolic prediction and therapeutic optimization, they should currently be regarded primarily as decision-support and hypothesis-generating systems until large-scale prospective multicenter studies establish their reproducibility, robustness, and sustained clinical benefit [36].

### 7.3. Multi-Kingdom Dynamic and Systemic Effects

The analysis of T2DM extends beyond bacteria to the broader multi-kingdom environment, specifically the gut virome and mycobiome. Phage-based interventions and virome transplantation are being explored as precision ecological tools to selectively deplete proinflammatory or metabolically deleterious bacterial populations. In parallel, mycobiome-targeted approaches, including probiotic supplementation with *S. boulardii*, have shown promise for enhancing intestinal barrier integrity and insulin sensitivity [47,48].

### 7.4. Integrative Frameworks: AI, Multi-Omics, and Digital Twins

The convergence of AI and multi-omics integration is transforming microbiome research from descriptive ecology to predictive systems biology. Notably, early digital twin clinical implementations have achieved reductions in hemoglobin A1c of up to 1.8% and reported T2DM remission rates approaching 73%, highlighting their translational potential in precision endocrinology [49,50]. However, the current evidence remains limited by the predominance of small-scale, single-center, and short-term studies, restricting the generalizability of reported findings [38]. No adequately powered multicenter randomized controlled trial has yet demonstrated superiority of microbiome-integrated digital twin systems over standard glycemic management.

Similarly, AI and machine-learning models trained on microbiome datasets often exhibit reduced predictive performance during external validation across geographically and clinically diverse cohorts [51,52]. Reported high predictive accuracies may partly reflect overfitting to cohort-specific microbial signatures, an issue compounded by the high dimensionality and limited sample sizes characteristic of microbiome studies [53]. Furthermore, many AI-based microbiome prediction frameworks rely on proprietary analytical pipelines and lack prospective clinical validation, underscoring the need for standardized methodologies, multi-cohort benchmarking, and rigorous external validation prior to clinical implementation.

By linking microbial taxonomic variation to functional outputs, contemporary analytical pipelines identify signatures predictive of insulin resistance and therapeutic responsiveness [29]. Recent multi-omics studies demonstrate that microbially derived metabolites can influence chromatin remodeling in hepatocytes and adipocytes, modulating the transcriptional control of glucose-handling genes [54,55,56]. Table 3 provides a comparative overview of emerging integrative frameworks combining multi-omics analysis, AI-driven prediction, and digital-twin technologies for precision management of T2DM.

### 7.5. Synthetic Biology and CRISPR-Based Platforms

Recent advances in synthetic biology and microbial engineering have transformed the gut microbiome from a passive therapeutic target to an active programmable treatment platform. Therapeutic strategies are no longer confined to conventional probiotic supplements. Instead, a new generation of engineered microbial consortia, programmable probiotics, and CRISPR-based delivery systems has emerged. These next-generation modalities are designed to dynamically sense and modulate the metabolic, immunological, and microbial network nodes that are implicated in the pathophysiology of T2DM.

By integrating synthetic gene circuits, biosensing modules, and targeted microbial editing, these platforms enable context-dependent therapeutic responses, adjusting microbial function in real-time based on host metabolic or inflammatory cues. When combined with digital co-therapies, such as AI-guided dosing algorithms and microbiome digital twins, these interventions establish closed-loop systems capable of continuous therapeutic optimization. Collectively, these advances position engineered microbiome therapeutics as adaptive and precise tools that complement pharmacological and lifestyle interventions in T2DM management.

#### 7.5.1. Engineered Probiotics and Synthetic Consortia

Recent advances have highlighted the application of engineered probiotics and synthetic microbial consortia as promising next-generation interventions for the management of T2DM. Emerging reviews have highlighted how genetically engineered probiotic strains can be designed to enhance intestinal barrier integrity, modulate inflammatory signaling pathways, and improve host glucose homeostasis through targeted metabolic outputs [34]. Engineered microbes can be programmed to secrete bioactive metabolites, reinforce mucosal immunity, or dynamically respond to host metabolic cues, thereby extending the capabilities of conventional probiotic formulations.

Complementary to single-strain approaches, synthetic microbiota strategies employ minimal, well-defined microbial consortia that recapitulate essential gut metabolic functions. By rationally assembling microbial communities with complementary enzymatic capacities, such consortia can be tailored to enhance SCFA biosynthesis, regulate bile acid transformation, or modulate host signaling pathways implicated in insulin resistance and metabolic inflammation [20]. Collectively, these approaches underscore the therapeutic potential of microbial ecosystems designed with precision, as modular and controllable interventions for T2DM.

#### 7.5.2. CRISPR-Based Microbiome Editing

Beyond the introduction of engineered microbial strains, a rapidly emerging frontier in microbiome therapeutics involves in situ editing of the native gut microbiota using CRISPR-based systems delivered by bacteriophages or synthetic vectors. Recent reviews have highlighted how CRISPR/Cas technologies can be programmed to selectively target microbial genes implicated in metabolic dysfunction, suppress pathogenic traits, and enhance beneficial metabolic pathways without disrupting the overall community structure [57,58]. Such precision editing enables functional modulation at the gene level, offering a level of control that surpasses that of the conventional probiotic or antibiotic strategies.

In parallel, phage-based interventions leverage the inherent host specificity of bacteriophages to reshape microbial populations and metabolic outputs with minimal off-target effects. Phage therapy approaches have been explored for their capacity to selectively deplete pro-inflammatory or LPS-producing bacteria associated with systemic inflammation and insulin resistance in T2DM [59]. The convergence of phage tropism with CRISPR precision establishes a platform for programmable, ecosystem-level microbiome engineering, laying the groundwork for next-generation precision microbiome therapies for metabolic diseases.

#### 7.5.3. Therapeutic Targeting of Microbial Ecosystem Networks

Contemporary microbiome engineering strategies are increasingly shifting from single-strain interventions to modulation of microbial ecosystem networks that govern metabolic homeostasis. Rather than focusing on individual taxa, these approaches target functional network nodes, including butyrate-producing communities, bile acid–modifying microbes, and indole-generating taxa that exert disproportionate control over host insulin sensitivity, inflammatory tone, and energy metabolism. Recent reviews of synthetic biology-based gut microbiome restoration have described how metabolic engineering can be leveraged to rewire microbial metabolic fluxes and redirect community-level outputs toward pathways that enhance insulin responsiveness and glycemic stability [60].

**Figure 4:**
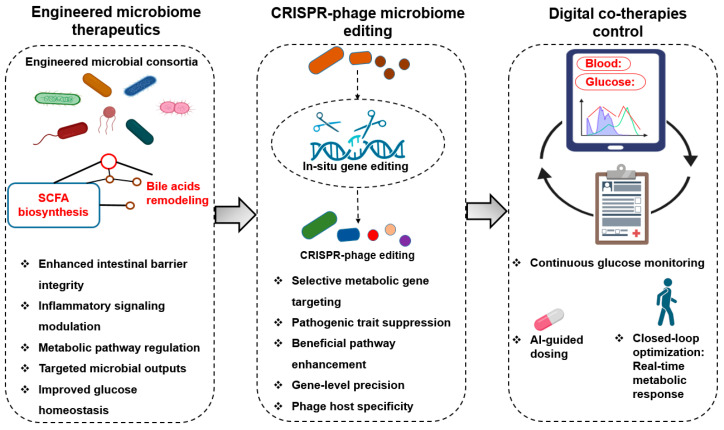
Schematic representation for precision management of T2DM through engineered microbial consortia, CRISPR–phage microbiome editing and digital co-therapy control. Arrows indicate the integration and progression of these approaches toward personalized T2DM management. Created with BioRender.com.

Beyond their role as adjuncts to conventional pharmacotherapy, engineered microbial networks are increasingly being envisioned as co-therapeutic systems that dynamically interface with digital health platforms. When coupled with continuous glucose monitoring, dietary tracking, and AI-driven feedback algorithms, network-targeted microbiome interventions could enable adaptive closed-loop modulation of host microbiome metabolism. This system-oriented paradigm underscores the potential of microbiome ecosystem engineering as a cornerstone for next-generation precision therapies for metabolic diseases. Figure 4 presents a graphical representation of a precision microbiome-centric model for T2DM management in which synthetic microbial consortia and CRISPR-based microbiome editing are integrated with digital health tools to enhance therapeutic specificity, minimize systemic dysbiosis, and enable adaptive co-therapeutic strategies tailored to individual patient profiles.

#### 7.5.4. Challenges and Clinical Translation

Despite their compelling mechanistic rationale, multiple barriers must be overcome before microbiome-based engineering strategies can be routinely deployed in clinical practice to treat metabolic diseases. The foremost among these are the safety considerations associated with engineered microbes and gene-edited strains, including the risks of horizontal gene transfer, off-target effects, and unintended perturbations of microbial ecosystem stability [35]. Robust biocontainment strategies, kill-switch designs, and long-term safety monitoring are essential to mitigate these concerns. Regulatory pathways for live biotherapeutic products and gene editing technologies remain under active development, creating uncertainty regarding approval frameworks, quality control standards, and post-marketing surveillance [61].

In parallel, the clinical evidence for T2DM remains limited, with a paucity of large-scale, randomized, and longitudinal trials capable of establishing durable efficacy, safety, and disease-modifying potential [62]. Additional translational challenges include ensuring manufacturing reproducibility, maintaining strain viability and functional stability during formulation and delivery, particularly within an anaerobic and spatially heterogeneous gut environment, and achieving sustained engraftment or functional persistence in the presence of host and microbial competition. Finally, the cost–benefit profile, scalability, and real-world effectiveness of these advanced therapies must be demonstrated across diverse patient populations to justify their integration into routine metabolic care.

Specific delivery technologies are being developed to address these barriers. Encapsulation strategies include alginate-chitosan microbeads, multilayer biopolymeric coatings, enteric polymers, hydrogel matrices, microencapsulation, and nano-/micro-formulations designed to protect viable probiotics or phages during gastric transit and promote intestinal release [63,64]. For phage and CRISPR–phage systems, protective delivery is especially important because free phages may lose infectivity under acidic gastric conditions; recent chitosan- and alginate-based carrier studies support their use for improving phage stability and controlled release [65]. Before clinical translation, computational predictions should be validated through a stepwise pipeline. In silico predictions should first be tested in anaerobic culture, defined synthetic communities, fecal batch cultures, and human intestinal organoid or gut-on-chip systems [66,67]. Candidate interventions should then be evaluated in mammalian models of metabolic dysfunction, including diet-induced or diabetic rodent models, with larger animal models considered when formulation, delivery, or pharmacokinetic scale-up is required [68]. Key endpoints should include fasting glucose, oral glucose tolerance, insulin tolerance, HbA1c or fructosamine, insulin/C-peptide indices, CGM where feasible, stool engraftment, quantitative polymerase chain reaction (qPCR) or shotgun-metagenomic confirmation, SCFA/bile acid/tryptophan metabolomics, drug pharmacokinetic/pharmacodynamic exposure, toxicity, and adverse-event monitoring [69].

Histopathological and tissue-level inflammatory validation should be incorporated when multi-kingdom interactions are proposed to influence systemic inflammation. Recommended readouts include hematoxylin and eosin (H&E) scoring of intestinal mucosal injury, goblet-cell and mucus-layer assessment, tight-junction protein staining, fluorescein isothiocyanate (FITC)-dextran permeability, liver steatosis/inflammation scoring, adipose macrophage infiltration, pancreatic islet morphology, and immunostaining or molecular quantification of tumor necrosis factor-alpha (TNF-α), interleukin-6 (IL-6), IL-1β, NF-κB activation, cluster of differentiation 68 (CD68) or F4/80-positive macrophages, and neutrophil markers [70,71]. These endpoints provide tissue-level evidence linking virome-mycobiome-bacterial perturbations to chronic metabolic inflammation rather than relying solely on fecal relative-abundance profiles [72].

For laboratory standardization, microbiome studies should adopt microbiome-specific reporting and technical standards, standardized sampling and storage, negative and positive controls, batch-randomized extraction, mock-community controls, harmonized sequencing pipelines, and explicit batch-effect assessment [73]. Absolute quantification by qPCR, droplet digital PCR (ddPCR), or flow cytometry should be used where possible to complement relative-abundance profiles [74]. The liquid chromatography-tandem mass spectrometry (LC-MS/MS) metabolomics should include pooled quality-control samples, analytical drift monitoring, and transparent quality-assurance procedures [75]. Advanced in vitro platforms should report oxygen gradients, medium composition, epithelial cell source, mucus production, microbial inoculum, multiplicity of infection or colonization, transepithelial electrical resistance (TEER) or barrier readouts, cytokine panels, and batch effects to improve reproducibility before moving to human cohorts [67].

## 8. Clinical Translation, Data Governance, and Regulatory Convergence

The translation of microbiome science into clinical practice is accelerating; however, regulatory, manufacturing, and implementation frameworks remain fragmented and incompletely aligned. As the gut microbiome transitions from a source of associative biomarkers to a therapeutic and diagnostic target in metabolic diseases such as T2DM, harmonized standards for safety, efficacy, manufacturing, and data integration are becoming increasingly essential to support scalable and reproducible clinical deployment.

### 8.1. Current Status of Clinical Implementation

Despite thousands of publications linking gut microorganisms to metabolic diseases, only a limited number of microbiome-based therapies have progressed to late-phase clinical trials or market authorizations [38]. This translational gap is particularly pronounced in T2DM, where the durability of microbiome-mediated effects, long-term metabolic benefits, and inter-individual variability in therapeutic responses remain insufficiently characterized. As a result, most microbiome-informed strategies for metabolic diseases are confined to the early-phase or proof-of-concept investigations. Table 4 presents an overview of microbiome-based therapies for T2DM, all of which are currently in the preclinical or Phase I/II stages of development [76,77,78,79,80,81].

### 8.2. Regulatory Frameworks for Microbiome-Based Therapies

Regulatory pathways originally designed for small-molecule drugs and monoclonal antibodies are poorly suited to emerging microbiome-based modalities, including live biotherapeutic products (LBPs), defined microbial consortia, bacteriophage therapies, and genetically engineered microbes. A comprehensive regulatory review outlines how European oversight is evolving under the regulation of Substances of Human Origin, while both the U.S. FDA and the European Medicines Agency (EMA) are developing microbiome-specific guidance documents [82]. Crucially, regulatory classification hinges on a product’s intended use (preventive, diagnostic, or therapeutic), underscoring the growing importance of regulatory science in establishing standardized criteria for quality, safety, potency, and clinical efficacy in microbiome-based interventions.

### 8.3. Translational Pipelines and Manufacturing Challenges

Effective clinical translation requires robust and standardized pipelines encompassing donor screening (for fecal microbiota transplantation or consortia-based approaches), strain-level characterization, functional potency assays, stability testing, and validated delivery platforms. In metabolic disease contexts such as T2DM, translational complexity is further amplified by dynamic host–microbiome–diet interactions, necessitating adaptive trial designs and real-world monitoring strategies. A persistent bottleneck remains the lack of validated analytical methodologies capable of quantifying microbiome functionality and therapeutic potency, which limits biomarker qualification and regulatory approval pathways [82]. Manufacturing reproducibility, batch-to-batch consistency, and regulatory traceability pose additional challenges, particularly for engineered or multi-strain microbial therapeutics.

### 8.4. Data Integration, Digital Health, and Ethics

As microbiome-guided therapies increasingly converge with digital health technologies, including CGM, AI-driven analytics, and wearable sensors, the regulatory scope expands to encompass data governance and ethical oversight. Key challenges include interoperability across platforms, protection of patient privacy, algorithmic transparency, and the governance of federated and longitudinal datasets. Recent analyses have emphasized that while microbiome diagnostics and interventions hold significant promise, their clinical translation is constrained by inconsistent data standards, regulatory uncertainty, and fragmented implementation pipelines [83]. In T2DM care, the integration of microbial, metabolic, and digital data streams demands coordinated oversight across medical device, pharmaceutical, and digital health regulatory domains.

### 8.5. Future Outlook and Recommendations

Realizing the full potential of microbiome-based precision endocrinology in T2DM requires a coordinated cross-sector action. Establishing harmonized international regulatory frameworks for LBPs, engineered microbial consortia, and phage-derived therapies is essential to ensure consistent quality, safety, and efficacy across jurisdictions.

In parallel, large-scale biomarker validation studies are required to link microbial signatures and metabolite profiles to therapeutic outcomes robustly, transforming microbiome-derived data into clinically actionable decision tools.

From a clinical development perspective, adaptive trial designs that account for dietary variability, baseline microbiome heterogeneity, and concomitant therapies should be prioritized along with the incorporation of digital twin and real-time feedback systems to optimize intervention strategies. Integration with digital health infrastructure, including wearable devices, cloud-based analytics, and AI-driven decision support, must proceed under stringent regulation-compliant data governance frameworks. Finally, the implementation of long-term post-marketing surveillance programs is critical for monitoring ecological stability, metabolic outcomes, and unintended effects, generating the longitudinal evidence required to refine both therapeutic strategies and regulatory policies [82,84].

## 9. Conclusions

The gut microbiome has undergone a profound conceptual transformation over the past decade, shifting from a descriptive biomarker of metabolic health to a programmable therapeutic organ. In T2DM, this shift is scientifically promising because microbial metabolism, intestinal barrier function, inflammation, and drug response intersect with core determinants of glycemic control. However, the revised evidence base also shows that microbiome–T2DM associations are heterogeneous, often medication-confounded, and not yet sufficiently validated for broad clinical deployment.

Despite the compelling potential of these advanced frameworks, significant hurdles must be overcome before they can be routinely deployed in clinical practice. Foremost among these are safety considerations regarding engineered microbes and gene-edited strains—such as the risks of horizontal gene transfer and unintended ecosystem perturbations—which necessitate the implementation of robust biocontainment and kill-switch designs. Furthermore, the field faces a lack of large-scale, randomized longitudinal trials capable of establishing durable efficacy and safety. These translational challenges are compounded by infrastructural barriers, including fragmented interoperability between wearable sensors, electronic health records (EHR), and microbiome analytical pipelines, which currently hamper scalability [37,41].

Addressing these gaps, alongside navigating emerging regulatory pathways—such as the FDA’s ongoing efforts to draft guidance on AI-based decision support tools and digital biomarkers for microbiome-targeted therapies—remains essential for clinical translation [56]. When these scientific, clinical, and regulatory pillars align, the gut microbiome will stand alongside classical metabolic organs as a central regulator of homeostasis. In the era of precision endocrinology, the gut will no longer serve as a static backdrop but will emerge as a dynamic control center—a programmable ecosystem guiding the future of evidence-based, adaptive metabolic medicine [83,85].

## Figures and Tables

**Table 1:** Key microbial taxa altered in T2DM and their metabolic impact.

Microbial Taxon	Alteration in T2DM	Principal Mechanistic Role	Clinical Relevance	References
*Faecalibacterium prausnitzii*	Decreased	Major butyrate producer; anti-inflammatory signaling; maintenance of epithelial barrier integrity	Reduced abundance correlates with insulin resistance, systemic inflammation, and disease severity	[1]
*Roseburia intestinalis*	Decreased	SCFA production; enhancement of gut barrier function	Loss associated with impaired barrier integrity and metabolic endotoxemia	[13]
*Akkermansia muciniphila*	Decreased at baseline; increased following metformin therapy	Mucin degradation; reinforcement of gut barrier; modulation of immune tone	Baseline abundance predicts metabolic health; enrichment associated with improved glycemic response to metformin	[15]
*Bifidobacterium adolescentis*	Decreased at baseline; enriched with anti-diabetic therapy	Carbohydrate fermentation; SCFA production; bile acid modulation	Associated with improved glucose tolerance and insulin sensitivity	[17]
*Escherichia coli–Shigella*	Increased	LPS production; induction of metabolic endotoxemia	Promotes systemic inflammation and insulin resistance; linked to disease progression	[1]

Abbreviations: LPS, lipopolysaccharide; SCFA, short-chain fatty acid.

**Table 2:** Multi-kingdom gut microbiota interactions and systemic effects in T2DM.

Microbial Component	Key Alterations in T2DM	Mechanistic Interactions	Host Pathways Affected	Metabolic and Clinical Consequences	References
Gut virome (bacteriophages)	Reduced viral richness and diversity; increased temperate phages targeting *Enterobacteriacea*	Phage-driven lysis and lysogeny reshaping of bacterial community structure and metabolic gene content; auxiliary metabolic genes modulate SCFA production and endotoxin release	TLR activation; nucleic acid sensing pathways	Enhanced metabolic endotoxemia, systemic inflammation, and insulin resistance	[29]
Eukaryotic enteric viruses	Increased presence of viruses such as noroviruses and enteroviruses in dysbiotic and metabolically unhealthy states	Viral infection induces mucosal inflammation and epithelial stress, compromising barrier integrity	Interferon signaling; cytokine networks	Mucosal inflammation and insulin resistance	[5]
Gut mycobiome (fungi)	Expansion of *Candida*, loss of beneficial fungi	Fungal antigens and cell-wall components activate innate immune signaling	Dectin-1 and NF-κB pathways	Dysbiosis associated with poor glycemic control	[3]
Fungi-bacteria crosstalk	*Candida* overgrowth with depletion of protective bacteria	Fungal metabolites suppress beneficial bacterial taxa and alter metabolite pools	Gut epithelial barrier	Inflammation, metabolic imbalance	[3]
Host-multi-kingdom immune crosstalk	Heightened sensing of microbial components across kingdoms	Viral, fungal, and bacterial cues amplify innate immune responses	TLRs, PRRs, cGAS-STING	Chronic inflammation and β-cell stress	[24]
System-level crosstalk	Coordinated multikingdom dysbiosys	Altered microbial metabolites and compromised barrier function	Gut-liver-brain axis	Disease progression and metabolic complications	[29]

Abbreviations: cGAS-STING, cyclic GMP–AMP synthase–stimulator of interferon genes; NF-κB, nuclear factor κB; SCFA, short-chain fatty acid; TLR, toll-like receptor; PRR, pattern recognition receptor.

**Table 3:** Comparative overview of multi-omics integration, AI predictive modeling and digital-twin ecosystems for microbiome-informed precision management of T2DM.

Feature	Multi-Omics Integration	AI Predictive Modeling	Digital-Twin Ecosystems	References
Primary objective	Mechanistic discovery and causal inference in host-microbiome-metabolome interactions	Prediction of glycemic trajectories and therapeutic response in T2DM	Personalized simulation and optimization of interventions	[29]
Core data types	Metagenomics, metabolomics, transcriptomics, epigenomics, longitudinal clinical phenotypes	Microbiome features, multi-omics data, CGM, BMI, insulin, clinical variables	Multi-omics, CGM, diet logs, microbiome dynamics, clinical history	[29,34,57]
Key analytical methods	Cross-platform integration, network analysis, pathway and regulatory mapping	Machine-learning models (e.g., LSTM, CNN, random forest, deep reinforcement learning)	AI-enabled dynamic modeling and iterative digital-twin simulation	[29,37,57]
Principal outputs	Microbial and metabolic signatures predictive of insulin resistance, glycemic trajectories, and therapy responsiveness	High-accuracy forecasts of glycemic variability and treatment response, with reported ROC values often >0.85	“What-if” scenarios for dietary, microbiome, and pharmacologic interventions	[29,41]
Barriers	Batch effects, data heterogeneity; and limited standardization across omics platforms; hinder reproducibility	Limited explain ability, reliance on proprietary APIs, and low integration into routine clinical workflows	Interoperability with EHR/wearables, regulatory uncertainty, privacy and security concerns, and lack of validated evaluation frameworks	[29,56]

Abbreviations: API, application programming interface; BMI, body mass index; CGM, continuous glucose monitoring; CNN, convolutional neural networks; EHR, electronic health record; LSTM, long short-term memory; ROC, receiver operating characteristic.

**Table 4:** Overview of microbiome-based therapies for T2DM currently in preclinical or Phase I/II stages of development.

Therapeutic Products	Target Indication	Development Stages	Mechanism of Action	References
AKK-WST01 (*Akkermansia muciniphila)*	T2DM, obesity	Phase II	Mucin-layer restoration, GLP-1 upregulation, barrier integrity reinforcement, metabolic endotoxemia reduction	[76,77]
MET-3 (multi-strain consortium)	T2DM, metabolic syndrome	Phase I/II pilot study	Metabolic-shifting agent, gut-derived inflammation reduction (endotoxemia), metabolic marker improvement	[76]
Xla1 (*Christensenella minuta*)	T2DM, metabolic syndrome, obesity	Early clinical trial Phase I	Promotes GLP-1 secretion, modulation of gut bile acid metabolism, enhancement of SCFA production, low-grade inflammation reduction	[78]
*Lactobacillus paracasei HII01*	T2DM	Phase II equivalent	Decrease fasting blood glucose, reduced inflammation (TNF-α, IL-6, hsCRP), ameliorated metabolic endotoxemia, improve hyperglycemia	[79]
*Lactobacillus paracasei IMC 502*	T2DM	clinical, animal (mice), and food fermentation contexts	Enhanced SCFA production, induce GLP-1 and PYY secretion, glucose tolerance	[80]
*Bifidobacterium lactis* V9-based LBP	Non-alcoholic fatty liver disease (NAFLD), T2DM	Preclinical/Animal mode	Modulation of gut microbiome, AMPK pathway activation, TLR-NF-κB inhibition (inflammation), reduced hepatic lipid accumulation, insulin sensitivity, lowered glucose level	[81]
*Lactobacillus plantarum* CCFM0236-based LBP	T2DM	Preclinical /academic	α-glucosidase inhibition, antioxidant modulation, inflammatory suppression, reduced TNF-α	[81]

Abbreviation: GLP-1, glucagon-like peptide-1; PYY, peptide YY; SCFA, short-chain fatty acid; TNF-α, tumor necrosis factor-α; IL-6, interleukin-6; hsCRP, high-sensitivity C-reactive protein; AMPK, AMP-activated protein kinase; T2DM, type 2 Diabetes Mellitus; TLR, toll-like receptor; NF-κB, nuclear factor-kappaB.

## Data Availability

No new data were created or analyzed in support of this research. This review article is based exclusively on data and information reported in previously published studies, appropriate copyright permissions and acknowledgments for reproduced or adapted figures have been provided where applicable.

## Abbreviations

The following abbreviations are used in this manuscript:

AI	Artificial Intelligence
AUROC	Area Under the Receiver Operating Characteristic Curve
CDC	Centers for Disease Control and Prevention
cGAS	Cyclic GMP-AMP synthase
CGM	Continuous Glucose Monitoring
CNN	Convolutional Neural Networks
CRISPR	Clustered Regularly Interspaced Short Palindromic Repeats
ddPCR	Droplet Digital PCR
FDA	Food & Drug Administration
FITC	Fluorescein Isothiocyanate
FXR	Farnesoid X Receptor
GEMs	Genome-Scale Metabolic Models
GLP-1	Glucagon-Like Peptide-1
GLP-1 RA	GLP-1 Receptor Agonist
GPR	G-Protein-Coupled Receptor
IDF	International Diabetes Federation
IL-6	Interleukin-6
LBP	Live Biotherapeutic Product
LPS	Lipopolysaccharide
LSTM	Long Short-Term Memory
MICOM	Microbial Community Modeling
NF-Κb	Nuclear Factor Κb
qPCR	Quantitative Polymerase Chain Reaction
RMSE	Root-Mean-Square Error
SCFA	Short-Chain Fatty Acid
SGLT-2	Sodium–Glucose Cotransporter 2
SHAP	SHapley Additive exPlanations
TEER	Transepithelial Electrical Resistance
TGR	Takeda G-protein Receptor
TLR	Toll-Like Receptor
TNF-α	Tumor Necrosis Factor-alpha
T2DM	Type 2 Diabetes Mellitus
